# A Numerical Analysis of Ductile Deformation during Nanocutting of Silicon Carbide via Molecular Dynamics Simulation

**DOI:** 10.3390/ma15062325

**Published:** 2022-03-21

**Authors:** Bing Liu, Xiaolin Li, Ruijie Kong, Haijie Yang, Lili Jiang

**Affiliations:** 1 School of Mechanical Engineering, Tianjin University of Commerce, Tianjin 300134, China; yongyuanreailxl@163.com (X.L.); 18535812170@163.com (H.Y.); 2 School of Mechanical Engineering, Tianjin University, Tianjin 300072, China; kongruijie@tju.edu.cn; 3 Tianjin Institute of Navigation Instrument, Tianjin 300131, China; 13920809662@163.com

**Keywords:** silicon carbide, molecular dynamics, nanocutting, machining mechanism, tool wear

## Abstract

As a typical third-generation semiconductor material, silicon carbide (SiC) has been increasingly used in recent years. However, the outstanding performance of SiC component can only be obtained when it has a high-quality surface and low-damage subsurface. Due to the hard–brittle property of SiC, it remains a challenge to investigate the ductile machining mechanism, especially at the nano scale. In this study, a three-dimensional molecular dynamics (MD) simulation model of nanometric cutting on monocrystalline 3C-SiC was established based on the ABOP Tersoff potential. Multi-group MD simulations were performed to study the removal mechanism of SiC at the nano scale. The effects of both cutting speed and undeformed cutting thickness on the material removal mechanism were considered. The ductile machining mechanism, cutting force, hydrostatic pressure, and tool wear was analyzed in depth. It was determined that the chip formation was dominated by the extrusion action rather than the shear theory during the nanocutting process. The performance and service life of the diamond tool can be effectively improved by properly increasing the cutting speed and reducing the undeformed cutting thickness. Additionally, the nanometric cutting at a higher cutting speed was able to improve the material removal rate but reduced the quality of machined surface and enlarged the subsurface damage of SiC. It is believed that the results can promote the level of ultraprecision machining technology.

## 1. Introduction

The third-generation semiconductor is considered a key material to support the rapid developments of new generation electronic information technology, such as 5G base station, new energy vehicle, fast charging, and other emerging industries. Silicon carbide (SiC), as one of the most significant third-generation semiconductor materials, has numerous advantages of large band gap, high thermal conductivity, high electron saturation drift rate, and large breakdown voltage intensity, and thus it is widely applied in the fields of smart grid, high-speed rail transit, armature system, and so on. Generally, in the practical industrial applications, the outstanding performance of SiC component can only be obtained when it has a high-quality surface and low-damage subsurface. However, due to the extremely low fracture toughness, high hardness, and brittleness, the surface quality of SiC component is difficult to dominate, which critically restrains its fabrication and application. Thus, the ductile machining mechanism of SiC with an ultrahigh smooth surface and low-damage subsurface at the nano scale is of important significance. Meanwhile, it is also a frontier topic with a great challenge.

Recently, nanometric cutting has been regarded as an effective means of ultraprecision machining technology [1,2,3,4], because it can directly realize the ductile removal of hard–brittle materials without fracture. Research showed that when the undeformed cutting thickness reduced to the nano scale, the ductile removal mode for the brittle materials was attainable and better surface quality could be obtained [5,6,7]. Therefore, using the nanometric cutting technology for ultraprecision machining of SiC ceramics improves the surface quality, enhances the machining efficiency, and substantially promotes the application of SiC semiconductor materials in various fields. However, the ductile machining mechanism SiC materials at the nano scale, including the mechanical behavior, deformation mechanism, and tool wear, is still unclear and needs to be studied in depth. For example, by experimental methods, Ding et al. [8] conducted both ultrasonic-assisted and conventional grinding experiments on the monocrystalline SiC; they discovered that the grinding force, surface roughness, and profile wave height were effectively reduced by the ultrasonic-assisted grinding. Pan et al. [9] performed systematic static stiffness indentation experiments on 6H-SiC ceramics, and machined the workpieces by utilizing fixed, free, and semi-fixed abrasives. Finally, they theoretically analyzed the nanomechanical properties and material removal mechanisms of SiC ceramics. Eswar et al. [10] investigated the mechanical response of monocrystalline SiC of two hexagonal polytypes (4H- and 6H-SiC) and studied the effect of crystal orientation on the material removal mechanism; they found a remarkable anisotropy that the 6H-SiC polytype exhibited a greater hardness than the 4H-SiC polytype.

Due to the expensive cost, time consuming tool setting, and high requirements for the machining environment with an experimental method, the molecular dynamics (MD) simulation method has attracted much attention for a growing number of scholars to study the machining mechanism of brittle materials, which can intuitively present the dynamic deformation behavior of the material atoms and greatly save the machining cost. Chavoshi and Luo [6] conducted nanometric cutting of single crystal silicon on different crystal orientations and at a wide range of temperatures (300–1500 K) through MD simulations; they found that smaller resultant force, friction coefficient at the tool/chip interface, and chip temperature was witnessed on the (1 1 1) crystal plane, as opposed to the other orientations. Shimada et al. [11] simulated the micro-indentation and cutting process on a non-defective surface; they pointed out that regardless of the inherent material properties, the ductile removal mode could be achieved if the machining feature size was small enough. Liu et al. [12] analyzed the scratching process of monocrystalline and polycrystalline SiC using a diamond tool by MD method; they found that the material removal process was achieved by the phase transformation to the amorphous structure both in the monocrystalline and polycrystalline SiC. Compared with monocrystalline SiC, less amorphous structure phase transformation, smaller normal scratching force, and higher tangential stress were discovered in polycrystalline SiC. Meng et al. [13] evaluated the diamond-machinability and removal mechanism of SiC-modified layer during femtosecond-laser-aided machining process using MD simulations. In addition, scholars have made lots of efforts on the generation mechanism of brittle–ductile transformation [14,15,16,17] and put forward various presumptions based on different theoretical methods, such as energy hypothesis [18], phase transformation [19], dislocation mechanism [20], phase transformation slip mixing mechanism [21], and stress distribution effect [22].

In summary, MD method can be considered as an effective means to study the material removal behavior at the nano scale. Thus, in this paper, multi-group MD simulations were performed to investigate the ductile machining mechanism of monocrystalline 3C-SiC. The main influencing parameters included the cutting speed and undeformed cutting thickness. The ductile machining mechanism, subsurface damage, cutting force, hydrostatic pressure, and tool wear are analyzed in depth. It is believed that the results can promote the level of ultraprecision machining technology.

## 2. Modelling

**Figure 1:**
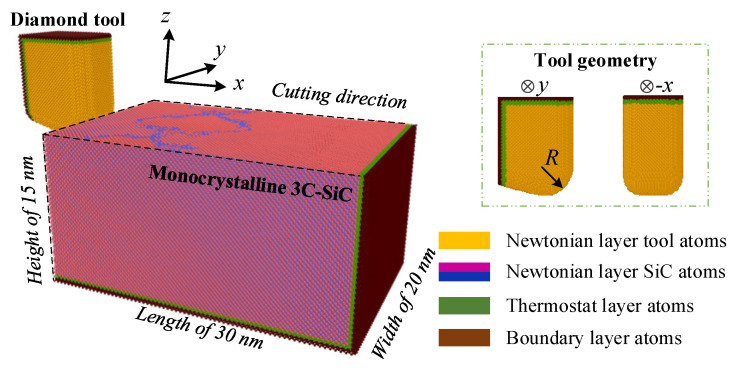
MD model of nanometric cutting on monocrystalline 3C-SiC.

The MD model of nanometric cutting on monocrystalline 3C-SiC was established as shown in Figure 1. The number of the tool and SiC atoms in the initial model were 86,000 and 882,221, respectively. The dimensions of the workpiece were 30 nm in length along the x direction, 20 nm in width along the y direction, and 15 nm in height along the z direction, respectively. Three parts of Newtonian layer, thermostat layer, and boundary layer were set in both the tool and SiC workpiece, ensuring that the simulated environment was closer to the actual machining condition. The interactions among the atoms in the Newtonian layer follows Newton’s second law by selecting an analytic bond-order potential (ABOP), which was successfully used in the previous literature [4,23]. The atoms in the thermostat layer were set to 300 K to simulate the heat dissipation in the actual environment, which further ensured the reliability of the simulation results. The atoms in the boundary layer were kept stationary to decrease the boundary effect. The nanocutting simulations were conducted along the <1 −2 1 0> orientation on the (0 0 0 1) crystal plane. To study the side flow behavior during the nanometric cutting process, the width of the SiC workpiece along the y-direction was set larger than that of the diamond tool, and the period boundary condition was adopted along the y-direction in all simulations. Note that the diamond tool was nonrigid in order to investigate the effect of nanometric cutting parameters on the tool wear, and further reflect the machinability of the SiC material. The canonical ensemble (NVT) was employed in the relaxation process to make the perfect initial lattice structure to be stable at the room temperature. Then, multi-group machining simulations were performed with various cutting speed and undeformed cutting thickness. The detailed nanometric cutting parameters in the MD simulations are listed in Table 1. All the subsequent visual observations were operated using the Open Visualization Tool (OVITO) [24], which can effectively analyze the ductile deformation during the nanocutting process.

## 3. Results and Discussion

### 3.1. Ductile Machining Mechanism

**Figure 2:**
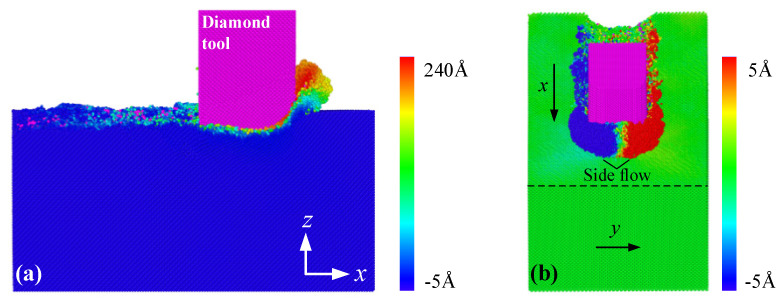
Variations of the atomic displacement vectors according to (**a**) x− and (**b**) y−direction.

**Figure 3:**
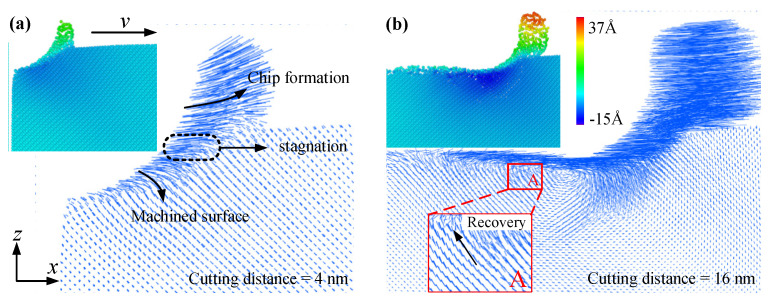
The displacement vectors of the workpiece atoms during nanometric cutting at a distance of (**a**) 4 nm and (**b**) 16 nm.

To study the ductile machining mechanism of 3C-SiC, the tool and workpiece atoms were colored according to their displacement vectors along the x- and y-direction, respectively, as shown in Figure 2. From the snapshot of x-z cross section in Figure 2a, the atomic accumulations were generated at the beginning of nanometric cutting under the extrusion action between the tool and the workpiece, and then a continuous chip was formed in front of the tool edge. The red atoms were the first parts to contact the tool, and the pink atoms behind the tool were the worn off parts which belonged to the tool. From Figure 2b, partial workpiece atoms flowed laterally, resulting in an apparent ploughing phenomenon. To clearly reveal the orientations of atomic flow, the displacement vectors of the workpiece atoms were presented in Figure 3, where all the atoms were concealed but only the arrows remained. The magnitude and direction of the blue arrows represented the absolute value and flow direction of atomic displacements, respectively. It can be found from the x-z sectional snapshot in Figure 3a that when the cutting distance was 4 nm, the workpiece atoms exhibited three different motion states: atoms moved upwards to form a chip, downwards to form a machined surface, and forward to form a stagnation region. When the cutting distance reached 16 nm, more atoms moved upwards, and the absolute value of atomic displacements increased. It can be seen from region A in Figure 3b, some atoms behind the tool in the workpiece subsurface moved upwards, indicating that a certain extent of elastic recovery occurred on the machined surface, which showed a good agreement with the previous study [25].

**Figure 4:**
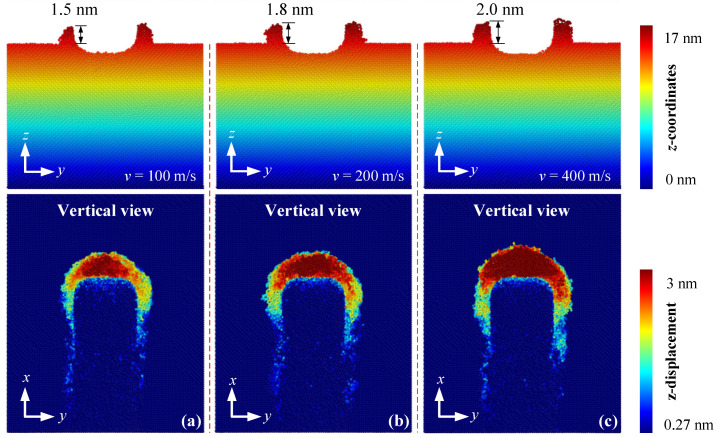
The lateral bulge and surface morphology of machined SiC at cutting speed of (**a**) 100, (**b**) 200, and (**c**) 400 m/s.

Figure 4 shows the effect of cutting speed on the machined surface morphology at a same undeformed cutting thickness of 2.5 nm. It is apparent that the height of lateral bulge at a high cutting speed was larger than that at a low cutting speed, indicating that the machined surface quality would be reduced by increasing the nanometric cutting speed, which was disadvantageous. From the vertical view in Figure 4, on the premise of the same cutting distance, the faster the nanometric cutting, the more workpiece atoms were removed. This indicated that a higher cutting speed can effectively improve the SiC material removal rate. The nanometric cutting at a higher cutting speed could reduce the cutting force and raise the workpiece temperature, which further made the material atoms easier to be removed.

**Figure 5:**
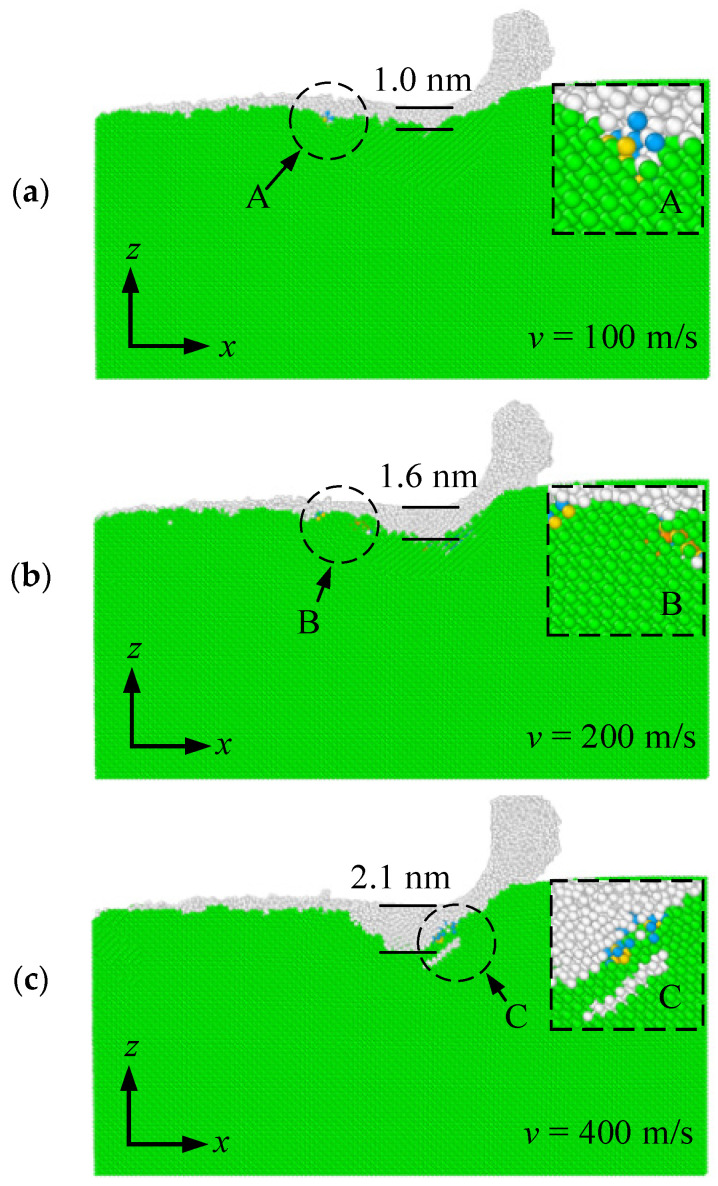
The effect of cutting speed on the subsurface damage at different cutting speed of (**a**) 100, (**b**) 200, and (**c**) 400 m/s.

In general, the subsurface quality can also affect the service performance and life of the components. Thus, it is particularly important to analyze the machining-induced damage of SiC and optimize the nanometric machining technology. Figure 5 shows the effect of cutting speed on the subsurface damage at a cutting distance of 16 nm, where the undeformed cutting thickness was 2.5 nm. Note that the green atoms represented the atoms with diamond structure and their neighbor atoms. The white atoms represented the amorphous atoms formed due to the lattice structure destruction caused by the extrusion action between the tool and the workpiece. The atoms in other colors represented the dislocations. It can be found that a certain extent of dislocation was produced at each cutting speed, as seen in the regions A, B, and C. With the increase of cutting speed, the number and length of dislocations decreased gradually. This is because with a higher cutting speed, the dislocations were removed in the form of chips before the evolution and reaction of dislocations could expand. This result is consistent with the previous study by Guo et al. [26]. Moreover, the thickness of the subsurface damage layer was measured to comparatively analyze the effect of cutting speed on it. The thickness of the subsurface damage layer increased with the increase in cutting speed. The temperature in the cutting zone rose with the increase in cutting speed, thus a high temperature can promote the amorphization generation of workpiece atoms. When the cutting speed was 400 m/s, the measured thickness of subsurface damage layer was approximately 2.1 nm, which was close to the undeformed cutting thickness.

**Figure 6:**
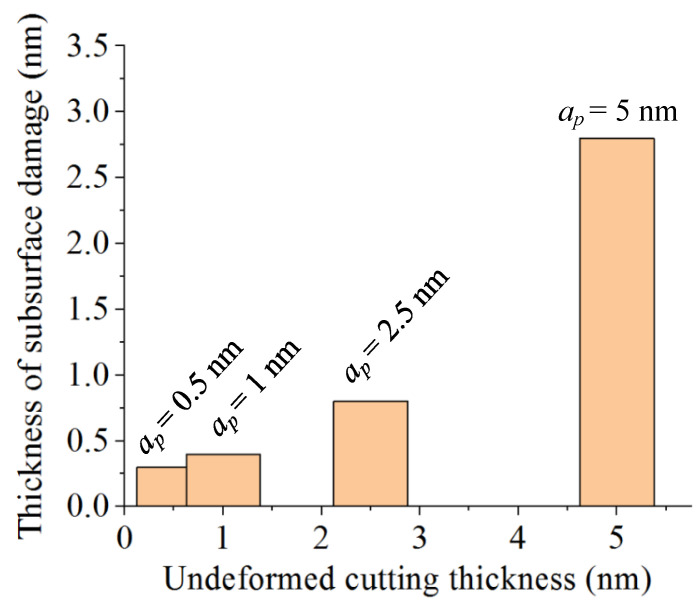
The variations of the subsurface damage with different undeformed cutting thicknesses.

Four groups of MD simulations with undeformed cutting thicknesses of 0.5, 1.0, 2.5, and 5.0 nm were performed to study its effect on the subsurface damage of monocrystalline SiC. The MD snapshots, which were similar to that in Figure 5, were no longer showed in this article to avoid redundancy. Figure 6 shows the variations of the subsurface damage with different undeformed cutting thicknesses, where the cutting speed was 400 m/s. The thickness of subsurface damage layer of SiC was positively correlated with the undeformed cutting thickness. When the undeformed cutting thickness was 5.0 nm, the thickness of the damaged layer was the largest, which was approximately three times that with a cutting thickness of 2.5 nm, and seven times that with a cutting thickness of 1.0 nm. In addition, nanometric cutting with a larger undeformed cutting thickness can result in more dislocation atoms. That is because the increase of the undeformed cutting thickness made the cutting force and cutting temperature greatly increased, leading to the lattice structure damage of the workpiece at the bottom of the tool, so that more amorphous atoms were generated.

### 3.2. Cutting Force and Hydrostatic Pressure

**Figure 7:**
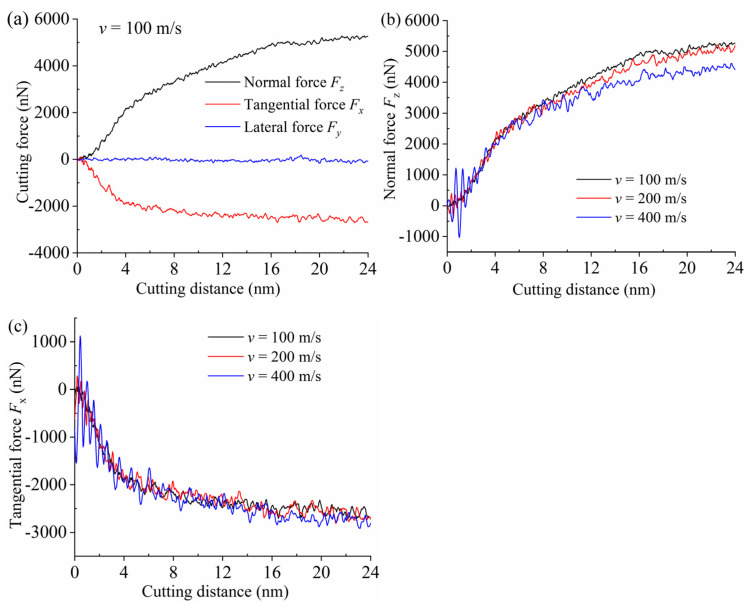
The analysis results of cutting forces at different cutting speed. (**a**) Cutting force; (**b**) Normal force; (**c**) Tangential force.

Figure 7a shows the analysis result of cutting force during the nanometric cutting at a cutting speed of 100 m/s. The force direction was consistent with that in Figure 1. In the beginning, with the increase in cutting distance, both the tangential force Fx and the normal force Fz increased due to the increase of the workpiece atoms accumulated on the tool rake face. Note that the Fx was negative because it was opposite to the cutting direction. The absolute value of Fx was generally smaller than that of Fz, which was reasonable for nanometric cutting with a large negative rake angle, especially when the undeformed cutting thickness was close to or even smaller than the tool edge radius [3,27]. The Fx tended to be stable when the cutting distance exceeded 8 nm, indicating that the tool completely cut into the workpiece at this time. When the cutting distance reached 16 nm, the Fz also tended to be stable, meaning that the number of piled-up atoms no longer increased, and partial workpiece atoms accumulated to a certain height to form a stable and continuous chip. In addition, because the opposite forces on both sides of the tool canceled each other, the lateral force Fy always fluctuated around zero. To reveal the effect of cutting speed on the cutting force, the analysis results of normal force Fz and tangential force Fx are presented in Figure 7b,c, respectively. Overall, the higher the cutting speed was, the smaller the Fz was. One reason is that a higher cutting speed can result in a higher cutting temperature of the workpiece, which was able to soften the SiC material and weaken the machining resistance. Another reason is that the higher the cutting speed was, the shorter the interaction time between the workpiece and the tool atoms was, which resulted in a smaller normal force. However, with the increase of cutting speed, the fluctuation of normal force after stabilization became violent, because the number of atoms interacting per unit time greatly increased, and the normal force fluctuation caused by covalent bond fracture needed to be completed in a shorter time. On the other hand, the Fx curves at three cutting speeds almost coincided during the whole cutting process. Since it was affected by the size effect, the normal force was dominant rather than the tangential force during the nanocutting process, therefore, the influence of cutting speed on cutting force was mainly reflected in the normal force. The results showed that a higher cutting speed can effectively reduce the normal force Fz, but it had little influence on the tangential force Fx.

**Figure 8:**
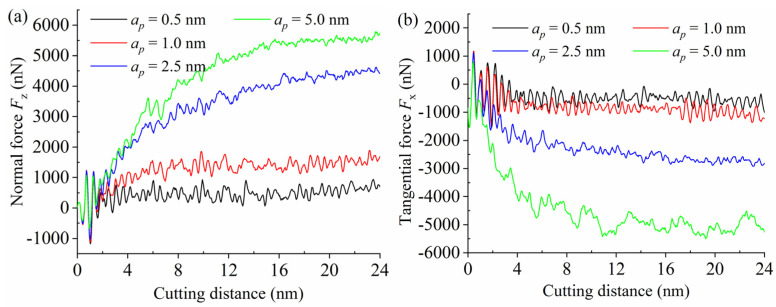
The variations of (**a**) normal force Fz and (**b**) tangential force Fx with different undeformed cutting thicknesses.

Figure 8 shows the effect of undeformed cutting thickness on the Fz and Fx at a same high cutting speed of 400 m/s. It is apparent that the undeformed cutting thickness had a greater impact compared with the aforementioned cutting speed. Both the Fz and Fx increased with the increase of the undeformed cutting thickness. This is because the contact area between the tool rake face and the workpiece expanded, implying that the number of workpiece atoms to be removed increased accordingly, so that a larger cutting force was necessary to resist the interatomic interactions. When the undeformed cutting thickness was 5 nm, the Fz and Fx were ultimately stable at approximately 5700 and 5100 nN, respectively. The fluctuation extent of the Fx curve was relatively great, as a result of the hard–brittle characteristic for SiC material. During the nanometric cutting process, the material removal mode was not simply ductile deformation or brittle fracture, as the above two modes usually appeared simultaneously. Moreover, accompanied by the breaking and recombination of covalent bonds, the shearing slip and lattice distortion always existed in the material removal process, which can temporarily reduce or enlarge the cutting force. It is worth noting that when the undeformed cutting thickness was 0.5 and 1.0 nm, the Fz and Fx curves gently increased and quickly reached a state of dynamic equilibrium. Under the condition of such a small undeformed cutting thickness, the workpiece emerged only elastic deformation without chip formation, namely, less atoms were accumulated in front of the tool edge even with no pile-up was generated [2].

**Figure 9:**
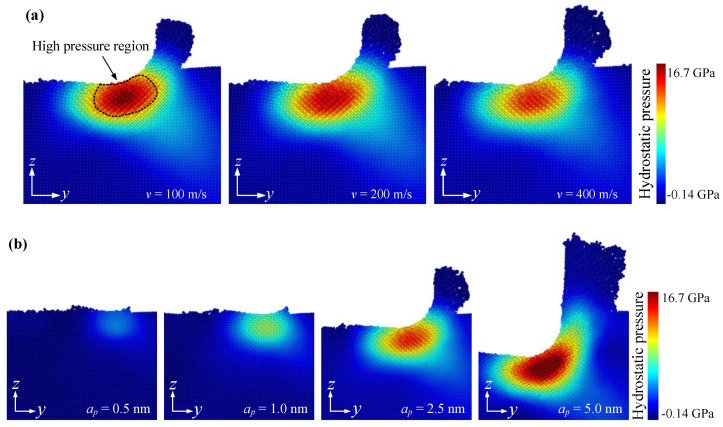
The effects of (**a**) cutting speed and (**b**) undeformed cutting thickness on the hydrostatic pressure during nanometric cutting.

Figure 9 shows the effects of cutting speed and undeformed cutting thickness on the hydrostatic pressure during nanometric cutting. It is found that due to the effective negative rake angle in nanometric cutting, an elliptical high-pressure region was generated under the interaction action between the tool and the workpiece, indicating that the chip formation did not strictly follow the traditional shear removal theory [28], but was dominated by the extrusion action. Additionally, the hydrostatic pressure of SiC material could be effectively reduced both by increasing the cutting speed and decreasing the undeformed cutting thickness, which was advantageous to improve the ductility of SiC and reduce the possibility of brittle fracture. This result was consistent with the analysis of cutting force discussed above, which verified the accuracy and reliability of the MD simulations.

### 3.3. Tool Wear

**Figure 10:**
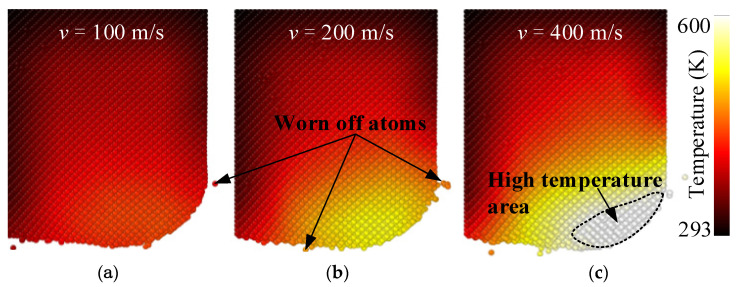
The effect of cutting speed on the temperature distribution of the diamond tool. The cutting speed was (**a**) 100 m/s, (**b**) 200 m/s and (**c**) 400 m/s, respectively.

In general, there is a close relationship between the tool temperature and the tool wear. Thus, the temperature distribution of the diamond tool at different cutting speed was analyzed, as shown in Figure 10. The maximum temperature of the tool was concentrated at the transition arc between the tool rake face and clearance face, namely the cutting edge. This phenomenon was obviously distinct from that in the nanometric cutting of ductile material, where the temperature distribution was uniform. The contact length between the tool rake face and the workpiece become longer with the enhancement of the material plasticity. For the brittle material SiC, the cutting heat was centered on the cutting edge and extended to the inside of the tool in a fan shape and gradually decreased. Overall, the faster the cutting speed, the higher the tool temperature. High cutting speed resulted in much cutting heat [29], which was not prone to be dissipated in time and finally affected the tool wear.

**Figure 11:**
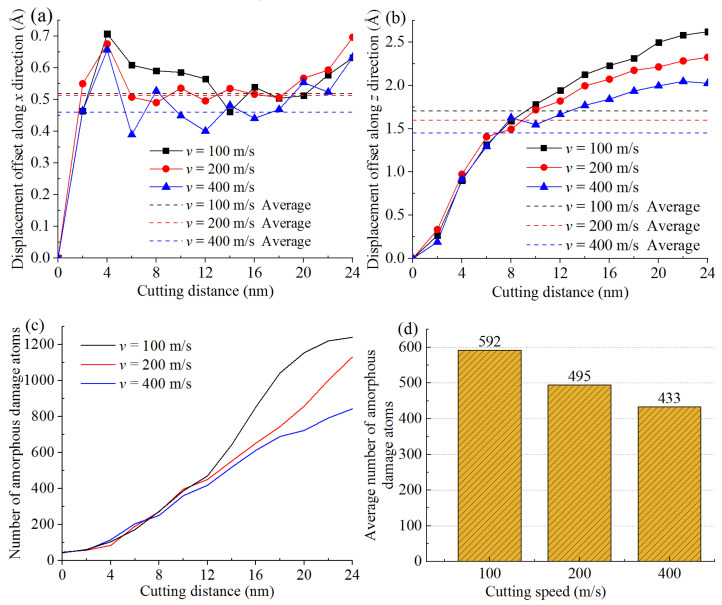
The analysis results of the tool wear at different cutting speed. Displacement offset along (**a**) x and (**b**) y direction; (**c**) Number of amorphous damage atoms; (**d**) Average number of amorphous damage atoms.

To quantitatively reveal the influence factors of tool wear, the effect of cutting speed was first studied and the corresponding result is shown in Figure 11. The curves in Figure 11a,b illustrated the variations of displacement offset between the used non-rigid tool and the ideal rigid tool along the x and z directions, respectively. First, the initial position of the tool was regarded as the coordinate zero point, then the displacement absolute values of all the non-rigid tool atoms were counted with the change of cutting distance. Next, the average displacement of all non-rigid tool atoms was subtracted from that of the ideal rigid tool. Afterwards, the displacement offset along the x and z directions was obtained, which can reflect the tool wear to a certain extent. At the beginning, the displacement offset at different cutting speed showed an increasing trend. As the cutting proceeded, the atoms in contact between the tool and the workpiece kept growing and the extrusion action increased accordingly. In this case, some of the tool atoms fell off or deviated from the proper position due to the chemical bond fracture. At this stage, the effect of cutting speed was inapparent. When the cutting distance reached approximately 8 nm, the tool completely cut into the workpiece, then the effect of cutting speed on the displacement offset became apparent. The results in Figure 11a,b also indicated that the higher the cutting speed was, the smaller the average displacement offset was, which reflected slighter tool wear. Therefore, in the scope of this study, the performance and service life of the diamond tool can be effectively improved by properly increasing the cutting speed. The reason is that a high cutting speed can result in a high workpiece temperature, which was able to soften the workpiece and made the workpiece atoms more conducive to be removed. Additionally, as aforementioned in Section 3.2, the cutting force would decrease with an increase in cutting speed, which also had a positive effect on reducing or restraining the tool wear. Nevertheless, this result was contrary to the machining theory in the conventional cutting, where the faster the cutting speed, the more serious the tool wear [30]. Consequently, to verify the reliability of the above results, the tool wear was further studied by the number of amorphous damage atoms in the tool during the whole nanometric cutting, as shown in Figure 11c,d. The effect regularity of cutting speed on the number of amorphous damage atoms was basically consistent with that in Figure 11a,b, which verified the reliability of the above results.

**Figure 12:**
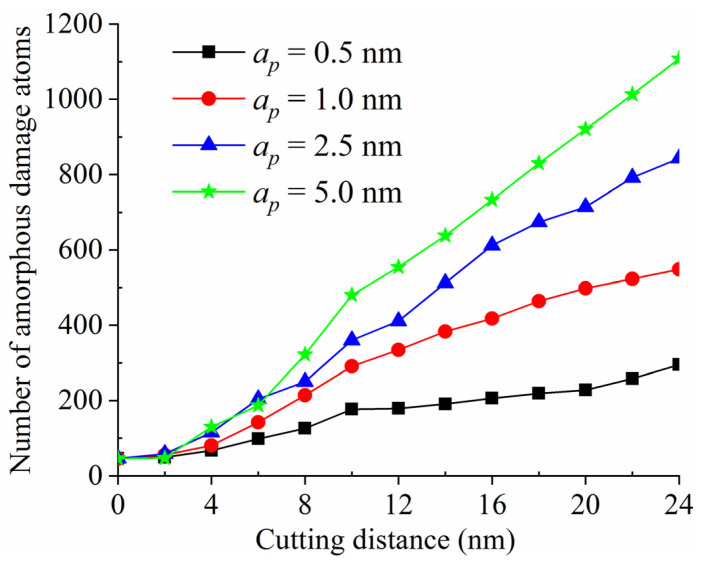
The variations of number of amorphous damage atoms with different undeformed cutting thicknesses.

Figure 12 shows the variations of the number of amorphous damage atoms with different undeformed cutting thicknesses. As the nanometric cutting proceeded, the number of amorphous damage atoms in the tool gradually increased, resulting in growing tool wear. In addition, the larger the undeformed cutting thickness, the more severe the tool wear under the premise of the same cutting distance. More atoms needed to be removed when the undeformed cutting thickness was large, leading to a greater force on the tool. Hence, the tool atoms were easier to deviate from the position they should be or fell off, and finally became the amorphous atoms.

## 4. Conclusions

In this study, a three-dimensional MD model of nanometric cutting on monocrystalline SiC was established based on the Tersoff potential, which could describe the deformation behavior under the extrusion action between the diamond tool and the SiC workpiece. Multi-group MD simulations were performed to investigate the removal mechanism of SiC at the nano scale. The effects of both cutting speed and undeformed cutting thickness on the material removal mechanism were considered. The ductile machining mechanism, cutting force, hydrostatic pressure, and tool wear was analyzed in depth. The main conclusions of this work are summarized below:

(1) Nanometric cutting at a higher speed was able to effectively improve the material removal rate, and reduce the cutting force and hydrostatic pressure, which were the favorable impacts. At the same time, it brought some adverse impacts, such as reducing the quality of machined surface and increasing the thickness of subsurface damage layer.
(2) In the nanometric cutting, the chip formation was dominated by the extrusion action rather than the shear theory. Nanometric cutting with a larger undeformed cutting thickness can improve the processing efficiency, however, it would enlarge the thickness of the subsurface damage layer, produce much more dislocation atoms, and increase the hydrostatic pressure.
(3) The maximum temperature of the tool was concentrated at the transition arc between the tool rake face and clearance face, which was obviously distinct from that in the nanometric cutting of ductile material. The performance and service life of the diamond tool can be effectively improved by properly increasing the cutting speed and reducing the undeformed cutting thickness.

## Figures and Tables

**Table 1:** Detailed nanometric cutting parameters in the MD simulations.

Contents	Detailed Parameters
Workpiece	Material: Monocrystalline 3C-SiC
	Dimensions: 30 × 20 × 15 nm^3^
Tool	Material: Nonrigid diamond
	Rake angle: 0°
	Clearance angle: 15°
	Edge radius: 2.5 nm
Cutting speed	100, 200, 400 m/s
Undeformed cutting thickness	0.5, 1.0, 2.5, 5.0 nm
Machining distance	0–24 nm
Nanocutting direction	<1 −2 1 0> orientation on (0 0 0 1) plane
Initial temperature	300 K
Thermostat method	NVT ensemble
Timestep	1.0 fs
Potential function	ABOP Tersoff potential
Integration algorithm	Velocity-Verlet

## Data Availability

Not applicable.
